# Serotonin Potentiates Transforming Growth Factor-beta3 Induced Biomechanical Remodeling in Avian Embryonic Atrioventricular Valves

**DOI:** 10.1371/journal.pone.0042527

**Published:** 2012-08-06

**Authors:** Philip R. Buskohl, Michelle L. Sun, Robert P. Thompson, Jonathan T. Butcher

**Affiliations:** 1 Department of Mechanical and Aerospace Engineering, Cornell University, Ithaca, New York, United States of America; 2 Department of Biomedical Engineering, Cornell University, Ithaca, New York, United States of America; 3 Department of Cell Biology and Regenerative Medicine, Medical University of South Carolina, Charleston, South Carolina, United States of America; Ecole Normale Supérieure de Lyon, France

## Abstract

Embryonic heart valve primordia (cushions) maintain unidirectional blood flow during development despite an increasingly demanding mechanical environment. Recent studies demonstrate that atrioventricular (AV) cushions stiffen over gestation, but the molecular mechanisms of this process are unknown. Transforming growth factor-beta (TGFβ) and serotonin (5-HT) signaling modulate tissue biomechanics of postnatal valves, but less is known of their role in the biomechanical remodeling of embryonic valves. In this study, we demonstrate that exogenous TGFβ3 increases AV cushion biomechanical stiffness and residual stress, but paradoxically reduces matrix compaction. We then show that TGFβ3 induces contractile gene expression (RhoA, aSMA) and extracellular matrix expression (col1α2) in cushion mesenchyme, while simultaneously stimulating a two-fold increase in proliferation. Local compaction increased due to an elevated contractile phenotype, but global compaction appeared reduced due to proliferation and ECM synthesis. Blockade of TGFβ type I receptors via SB431542 inhibited the TGFβ3 effects. We next showed that exogenous 5-HT does not influence cushion stiffness by itself, but synergistically increases cushion stiffness with TGFβ3 co-treatment. 5-HT increased TGFβ3 gene expression and also potentiated TGFβ3 induced gene expression in a dose-dependent manner. Blockade of the 5HT2b receptor, but not 5-HT2a receptor or serotonin transporter (SERT), resulted in complete cessation of TGFβ3 induced mechanical strengthening. Finally, systemic 5-HT administration *in ovo* induced cushion remodeling related defects, including thinned/atretic AV valves, ventricular septal defects, and outflow rotation defects. Elevated 5-HT *in ovo* resulted in elevated remodeling gene expression and increased TGFβ signaling activity, supporting our *ex-vivo* findings. Collectively, these results highlight TGFβ/5-HT signaling as a potent mechanism for control of biomechanical remodeling of AV cushions during development.

## Introduction

Biomechanical remodeling is the process by which living tissues reorganize, reshape, and refit their microstructure in adaptation to changing internal and external forces. This process defines much of embryogenesis, during which initially indistinct cellular masses acquire shape and functional specificity through production and manipulation of the extracellular matrix (ECM). This is particularly important for the morphogenesis of the heart, which is critically responsible for distributing nutrients as the embryo grows. The heart transitions rapidly from a tubular structure into a multi-chambered pumping organ, simultaneously growing over 100-fold in volume [1]. The hemodynamic environment inside the heart increases dramatically in severity during this process [2]–[4], which means the biomechanical properties of the forming valves must be precisely tuned to maintain efficient unidirectional blood flow. Atrioventricular (AV) valve morphogenesis is characterized by rapid ECM accretion and turnover [5], [6], which is hypothesized to be stimulated by a dynamic interaction of molecular and mechanical signaling. While numerous molecular agents important for valve morphogenesis have been identified [7]–[10], less is known about how these signals affect valve mechanics, which is a key readout of valve function.

The transforming growth factor-beta (TGFβ) superfamily is critically important for a wide range of cellular processes [11]–[13], and is heavily involved in directing morphogenesis of AV cushions [14]–[18]. In the chick, TGFβ2 and TGFβ3 isoforms are necessary for the endothelial to mesenchymal transition (EMT) which initiates AV cushion development [19]. TGFβ2 induces initial cell-cell separation of valve endothelial cells, while TGFβ3 stimulates their invasion and subsequent mesenchymal phenotype shift [15], [16]. During post-EMT, these mesenchymal cells facilitate a transition in the cushion microstructure from glycosaminoglycans (GAGs) (hyaluronan, versican) toward fibrous structural proteins (collagen I, IV, V, fibronectin, periostin) [5], [20], [21]. This shift in ECM content translates into increased valve stiffness [22], and coincides with elevated expression of TGFβ3 in the cushions and AV canal [23]. Furthermore, TGFβ3 upregulates collagen I and periostin in post-EMT AV cushion explants [24], suggesting that TGFβ3 is a key modulator of cushion ECM content, and consequent mechanical properties. An aim of this study is to better understand this remodeling potential of TGFβ3 through a combined analysis of cushion stiffness, matrix compaction, cell proliferation, and ECM synthesis.

The capacity of TGFβ3 to stimulate valvular remodeling events underscores the importance of identifying molecular signals which modulate TGFβ activity. Recent studies indicate that serotonin (5-HT) interacts with TGFβ signaling in adult heart valves [25], [26], and can also alter valve mechanical properties [27], [28]. 5-HT, which is a monoamine neurotransmitter derived from the essential amino acid tryptophan [29], increased the stiffness of porcine aortic valve cusps with the endothelial layer denuded [27], and under cyclic stretch [30]. Serotonin also increased collagen synthesis in human and sheep valve interstitial cells (VICs) [25], [31]. Reports in adult VICs indicate that 5-HT can upregulate TGFβ, resulting in cell differentiation and aberrant connective tissue accumulation [25], [26], [32]. In development, serotonin is active in key events such as cardiac progenitor patterning, left-right laterality, and migration of the neural crest [33]–[37]. Murine AV cushions express the serotonin receptors 5-HT2a and 5-HT2b, and the serotonin transporter (SERT) by the completion of EMT [38], [39], which is when TGFβ3 expression increases in the cushions [18], [40]. Latent TGFβ binding protein and serotonin binding protein are also expressed in murine post-EMT endocardial cushions [41], [42], highlighting each pathway's capacity to regulate expression of their ligands. The proximity of these TGFβ and 5-HT signaling components suggests that they may be interacting partners in post-EMT cushion development. Furthermore, a recent study reported TGFβ1 upregulation in murine SERT KO hearts at near fetal stages, which was hypothesized to be a consequence of excess 5-HT signaling due to SERT inhibition [43]. In light of these signaling interactions in both adult and development models, we hypothesize that this mechanically relevant crosstalk of TGFβ and 5-HT may play a role in modulating embryonic AV cushion biomechanics.

The objectives of this study therefore were to characterize the remodeling capacity of TGFβ3 in AV cushions, and determine how TGFβ3 and 5-HT may act together to regulate cushion biomechanical remodeling. Chick AV cushion biomechanics, compaction, and candidate gene expression were quantified through implementation of an *ex vivo* cushion culture system. We determined that TGFβ3 induces AV valve stiffening through increases in cell proliferation, myofibroblastic differentiation, and collagen synthesis. 5-HT enhances the AV valve stiffening effect of TGFβ3 in a dose-dependent manner. Crosstalk between TGFβ3 and 5-HT signaling was investigated via molecular inhibition studies. The *ex vivo* results were then tested *in ovo* through an elevated 5-HT model. These results suggest that 5-HT may be an important potentiator of TGFβ3 signaling in embryonic valve morphogenesis and biomechanical stiffening.

## Materials and Methods

### Ethics Statement

Leghorn avian embryos from Hamburger-Hamilton stages (HH) 17–36 were utilized in this research. All procedures in this study followed the guidelines of Cornell University and NIH policy, which state that avian embryos of these stages are not considered vertebrate animals for the purposes of IACUC regulation.

### AV cushion remodeling organ culture model

Fertilized leghorn chicken eggs were incubated until stage HH25 (Day 4.5). The AV cushions were isolated from their myocardial attachment in ice-cold sterile Earle's Balanced Salt Solution (EBSS; Quality Biological, Inc.). Single cushions were cultured in 20 µL hanging drops for 24 hours at 38°C in a 5% CO_2_ environment. Control culture media consisted of Medium 199 (M199 w/phenol red and L-glutamine; Gibco) with 1% concentrations of penicillin/streptomycin (Gibco), Insulin-Transferrin-Selenium (ITS, Gibco), and chick serum (Gibco). For experiments, control media was treated with one or more of the following reagents: human recombinant TGFβ3 (1 ng/ml, Sigma), serotonin hydrochloride (0.47–47 µM, Sigma), Cytochalasin D (1 µM, Sigma), 5-HT2a inhibitor MDL100,907 (0.01–1 µM, Axon Medchem BV), 5-HT2b inhibitor SB204741 (0.35–35 µM, Sigma), SERT inhibitor Fluoxetine (1–10 µM, Sigma) and Alk 4,5,&7 inhibitor SB431542 hydrate (0.26–26 µM, Sigma). TGFβ3 was reconstituted in 4 mM HCl solution containing 1 mg/ml BSA, all other reagents were dissolved in DMSO. The 470 nM 5-HT dose was considered physiological, based on HPLC measured concentrations in 10% fetal bovine serum media (∼100 nM [44]). The 47 µM 5-HT dosage is similar to prior *in vitro/ex vivo* studies in postnatal valves [25], [27], [28], [30], [45], so we conservatively considered this dose high for our studies.

### Micromechanical testing

**Figure 1:**
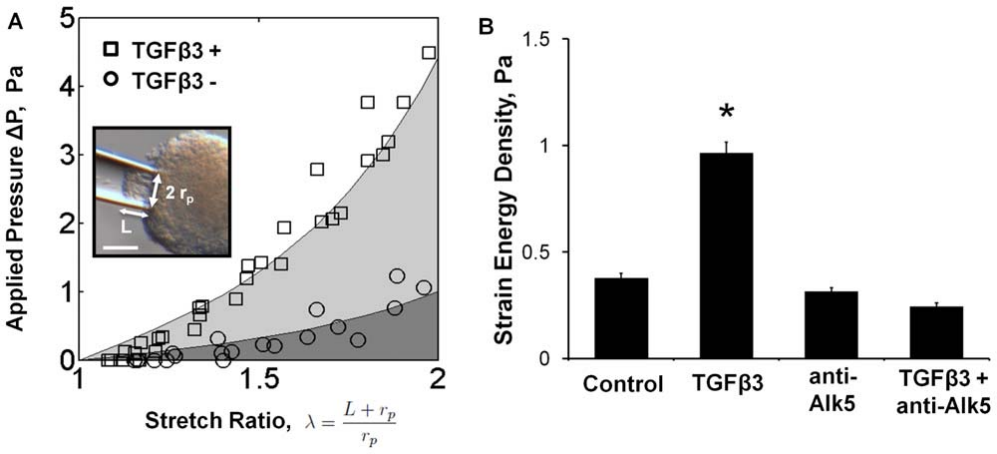
TGFβ3 treatment increases stiffness of AV cushions through Alk5 mediated pathway. **A**) Representative pipette test data for TGFβ3 (1 ng/ml, TGFβ3+) and control media (TGFβ3−) treated cushions, n = 4. Strain energy density was calculated from the shaded regions beneath the ΔP vs λ curves. Inset: image of aspirated HH25 AV cushion after 24 hours of culture. The pipette radius, r_p_, and the aspirated length, L are indicated. Scale bar = 70 µm. **B**) AV cushion strain energy density increased with TGFβ3 treatment, but was blocked by Alk5 inhibition (SB431542, 2.6 µM). mean ± SEM, n≥7, *p<0.0001, 2-way ANOVA.

Cushion mechanical properties were measured after 24 hour treatment in the *ex vivo* study and at HH25 in the *in ovo* study using the micromechanical pipette aspiration technique [22], [46], [47]. A glass micropipette (∼70–100 µm in diameter) was placed adjacent to the cushion surface, and a small vacuum pressure was incrementally applied. The pressure source was a 200 µL pipetter calibrated with a custom manometer. Previous strain history was mitigated by preconditioning with ∼20 cycles of low pressurization (<1 Pa). The tissue was then monotonically loaded with increasing static pressure loads, at which images were captured. Aspirated length L, measured as the length from the tip of the pipette to tip of the tissue furthest inside the pipette, was converted into an experimental “stretch ratio”, 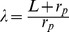, by normalizing to the pipette radius, r_p_. The cushion was assumed to be an isotropic, incompressible, hyperelastic material with an exponential free energy law, 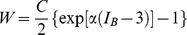, where I_B_ is the first invariant of left Cauchy Green stretch tensor. AV cushion material isotropy at HH25 was supported by a lack of preferred matrix orientation as determined by ubiquitous protein stain 5-DTAF (50 µM Invitrogen; Figure S1). The ΔP vs. λ data was then fit to the axial stress equation of a uni-axially loaded bar of this exponential material, specifically, 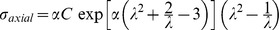. From previous analysis [22], the ΔP vs. λ curve differs from the uniaxial load expression by a scale factor, γ. This scale factor was numerically determined to be a function of only the material parameter α. Due to the nonlinear nature of the data, the mechanical testing data is presented as strain energy density. This was calculated as the area under the ΔP vs. λ curve fit from λ = 1–2 (Figure 1A), which from our assumed material model is 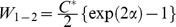, where C^*^ = γ C.

### Compaction & Opening Angle Assays

Compaction of the AV cushions was quantified as the ratio of cross-sectional area before (A_0_) and after (A) 24 hours of culture in the different treatment conditions, denoted A/A_0_. This ratio measures the combined biomechanical remodeling effects of cell traction, proliferation, and ECM synthesis. To isolate cell traction effects, we quantified the opening angles created by micro-slit incision in AV cushions after 24 hours of treatment. The incision was made along the centerline of the spherical cushion mass, extending approximately one radius into the cushion, and immediately created a pie-wedge with defined opening angle. Opening angles are an established indicator of tissue residual stress [48], which is primarily a function of cell traction forces in our culture system. Images were taken at 150× magnification using Zeiss Discovery v20 stereomicroscope (Spectra Services, Inc.) and QImaging Retiga 4000R Fast camera (Spectra Services, Inc). Cross-sectional area and opening angle were measured from calibrated images using NIH ImageJ image analysis software.

### Immunohistochemistry (IHC)

Proliferation was assessed through bromodeoxyuridine (BrdU) incorporation into HH25 AV cushion hanging drops. BrdU reagent (Invitrogen) was added at 1∶100 dilution in culture medium 6 hours prior to completion of 24 hour culture. AV cushions were then rinsed and fixed in 4% paraformeldehyde (PFA). BrdU incorporation was assessed via immunofluorescent antibody staining and confocal microscopy using anti-BrdU 488 (1∶100, Invitrogen), with DRAQ5 (1∶1000, Biostatus) as a DNA counterstain. Images were processed via ImageJ, and BrdU incorporation was quantified as the ratio of BrdU positive cells to total cell count. IHC was also used to label phosphorylated Smad2/3 (pSmad2/3) complex in HH25 cushions isolated from the systemic 5-HT *in ovo* model. Isolated cushions were fixed in 4% PFA and then stained via standard whole mount IHC protocol. The cushions were stained with primary pSmad2/3 polyclonal goat anti-human antibody (1∶50, Santa Cruz) followed with 488 fluorescent secondary (1∶100, Santa Cruz) and cell nuclei counter stain DRAQ5 (1∶1000). pSmad2/3 was quantified as the number of cell nuclei with localized pSmad2/3 divided by the total number of cell nuclei.

### PCR quantification of gene expression

At the end of 24 hour treatment, AV cushion mRNA was isolated and purified using RNEasy Isolation Kit (Qiagen). A set of 8–10 cushions were pooled per test sample. RNA integrity was determined by NanoDrop spectrometry, using A260/A280 ratio between 1.8 and 2.2 as quality control. cDNA synthesis was completed using SuperScript III first strand RT-PCR kit (Invitrogen) with oligo(dT) primers. Amplification reactions were as follows: (95°C 15 s), (54°C 15 s), (72°C 30 s). Power Syber Green (Applied Biosystems) replication indicator was read at the completion of each 72°C stage. Standard curves for all primers (listed in Table S1) were generated from HH34 brain mRNA and normalized to 18 s ribosomal RNA. Threshold cycle count, C(t), was used to calculate gene expression via the ΔΔCt method using 18 s rRNA as a housekeeping reference gene [49].

### 5-HT administration in ovo

HH17 stage fertilized leghorn chicken eggs were windowed on their blunt side. Up to 1.0 mg of serotonin (Sigma) was diluted into 100 µL of PBS and dispensed directly onto the chorionic membrane at HH17, HH25, or HH31. The max 5-HT dosage was equivalent to 18 mg/kg which is comparable to other elevated 5-HT animal models (25 mg/kg and 75 mg/kg) [50], [51]. After 5-HT treatment, chicks were then sealed and cultured at 55% humidity and 38°C until HH36 (Day 10). Preliminary experiments demonstrated that 5-HT treatment sometimes resulted in an ectopic heart, so additional embryos were alternatively subjected to a thoracotomy that mimicked an ectopic heart without serotonin administration as a control. Embryos were then dissected and analyzed for gross anatomical defects. Hearts with intact great artery connections were then removed, cleared, and analyzed with 3D confocal microscopy or serial section histology using Movat's pentachrome stain. Optical fluorescence tomography (OFT) of ventricular, valve, and outflow vessel anatomy was performed as previously described [52], [53]. Briefly, HH36 hearts were freshly isolated and rinsed with 1% lidocaine in PBS buffer. Following rinse, hearts were perfused with fluorescein isothiocyanate–poly-L-lysine (Sigma) via micro injection and then fixed in 4% PFA. The poly-L-lysine binds to the negatively charged endothelial glycocalyx. Hearts were then cleared using Murray's Clear, followed by deep tissue 3D imaging via fluorescence confocal microscopy. Hearts were screened for major defects, and valve morphometry were quantified from this using ImageJ. Valve measurements included leaflet length, average thickness, and minimal thickness with control n = 3 and 5-HT treatment n = 6. Average thickness (t_avg_) was calculated as t_avg_ = A_L_/L, where L is the annulus-tip length of the leaflet, and A_L_ is cross-sectional area of leaflet. The location of minimum thickness was generally the same for all specimens regardless of treatment.

### Statistical Analyses

All data is presented as mean ± standard error of the mean for the number of samples reported. Statistical comparisons between groups were performed using ANOVA for data sets involving more than two groups, or two-tailed t test when only two groups were compared. Defect prevalence in the *in ovo* model was compared using a chi-squared statistical test. In all comparisons, differences between groups was considered statistically significant for p valves smaller than 0.05.

## Results

### TGFβ3 increases AV cushion stiffness

**Figure 2:**
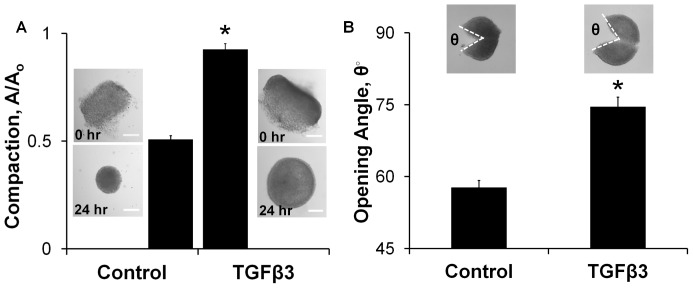
TGFβ3 treated cushions compact less than controls, but are under more residual tension. **A**) Bar graph of area ratios calculated from before and after images of 24 hour TGFβ3 treated cushions. Representative cushion images shown, scale bar = 100 µm. mean ± SEM, n≥12, *p<0.0001, t-test **B**) Opening angle of 24 hour TGFβ3 treated cushions is greater than control, indicating tissue is under greater residual tension. Inset shows representative images with opening angle, θ. mean ± SEM, n = 10–11, *p<0.001 t-test.

*Ex vivo* cultured AV cushions exhibited nonlinear mechanical behavior that was well described by the exponential constitutive model (Figure 1A). Administration of exogenous TGFβ3 (1 ng/ml) increased cushion stiffness 2.5 fold over controls (W_TGFβ3_ = 0.965±0.051 vs. W_Contr_ = 0.378±0.021, p<0.0001 Figure 1B). Inhibition of canonical TGFβ signaling via the TGFβ type 1 receptor Alk 5 (2.6 µM SB431542 [54]) blocked the increase in cushion stiffness (W_T+TI_ = 0.245±0.043 Figure 1B). The Alk5 inhibitor alone had no effect on cushion biomechanics. TGFβ3-treated cushions compacted less than controls, with compaction quantified as the ratio of cross-sectional area before and after treatment (A/A_0_ = 0.925±0.028 vs. A/A_0_ = 0.508±0.017, p<0.0001 Figure 2A). This was unexpected because the Cytochalasin D (CytD, 1 µM) results suggested that compaction and stiffness are directly related. CytD inhibited cytoskeletal actin polymerization which resulted in a 5.3 fold decrease in strain energy density of the AV cushions relative to control (W_CytD_ = 0.072±0.016, Figure S2A). Without actin polymerization the AV cushion cells did not compact the matrix, and the cushion did not remodel into the spherical configuration observed in all other treatments. Instead, the post-treatment cushion area was significantly larger than initial area, suggesting a relaxation of pre-treatment actin forces (A/A_0_ = 1.60±0.03, Figure S2B). The TGFβ3 results of stiffness increase with compaction decrease did not align with this trend. Alk5 inhibition did return compaction behavior to control levels (A/A_0_ = 0.570±0.035 Figure S3), indicating that the stiffness and compaction results are both dependent on activation of canonical TGFβ3 signaling. To better understand the relationship between stiffness and compaction, cushion opening angles were quantified to approximate differences in cell traction forces. The opening angle of TGFβ3 cushions was 1.29 fold larger than controls (74.6°±2.0° vs. 57.7°±1.4°, p<0.001 Figure 2B), indicating that TGFβ3 treated cushions did indeed have higher cell traction forces. Together, these results demonstrate that TGFβ3 induces cushion stiffening through Alk5, but with a concurrent reduction in tissue compaction that suggests other processes are also affected.

### TGFβ3 increases AV cushion proliferation and mesenchymal phenotype

**Figure 3:**
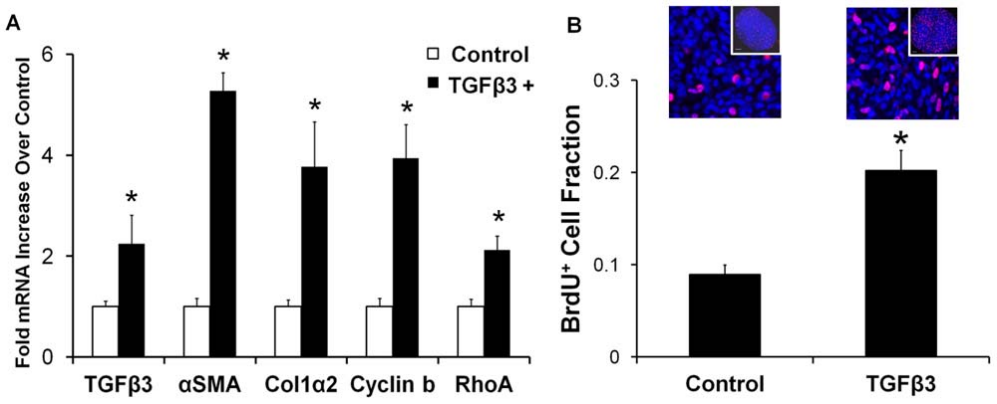
Remodeling behavior of TGFβ3 treated cushions is a balance of contractile differentiation, proliferation, and matrix synthesis. **A**) 24 hour TGFβ3 treated cushions upregulate contractile (αSMA, RhoA), proliferation (cyclin b), and extracellular matrix protein (col1α2) encoding genes. TGFβ3 administration also significantly stimulated its own production. mean ± SEM, n = 3–4 pooled samples of 8–10 cushions, *p<0.05, t-test. **B**) BrdU incorporation data (red) of TGFβ3 treated cushions normalized to DRAQ5 cell nuclei counter stain (blue). BrdU was administered 6 hours prior to completion of 24 hour treatment. Representative confocal images are shown above each bar, with a global view of cushion contained in the inset. mean ± SEM, n = 12, *p<0.0001, t-test.

Contractile phenotype markers αSMA and RhoA were significantly upregulated with TGFβ3 treatment, 5.3±0.4 and 2.1±0.3 fold (± SEM) respectively (Figure 3A), suggesting that TGFβ3 induced residual tension is partially due to an increased migratory/contractile phenotype of resident cushion mesenchyme. TGFβ3 treatment also upregulated mRNA expression of col1α2 mRNA (3.8±0.9, p<0.05) and cyclin b2 (3.9±0.7 fold, p<0.05), indicative of increased collagen I synthesis and cell proliferation, respectively. BrdU incorporation confirmed that TGFβ3 increased cushion cell proliferation 2.26±0.36 fold over controls (p<0.0001, Figure 3B). Collectively, these results strongly suggest that while TGFβ3 treated AV cushion mesenchyme are more migratory/contractile, concomitant increases in cell proliferation and matrix synthesis work to counteract aggregate matrix compaction. This explains how the TGFβ3 treated cushions are biomechanically stiffer, but appear minimally compacted. Furthermore, TGFβ3 treatment increased TGFβ3 transcription (2.2±0.6 fold, p<0.05), indicating a potential positive feedback loop for TGFβ3 control of AV cushion biomechanical remodeling.

### 5-HT potentiates TGFβ3 signaling through 5-HT2b receptor

**Figure 4:**
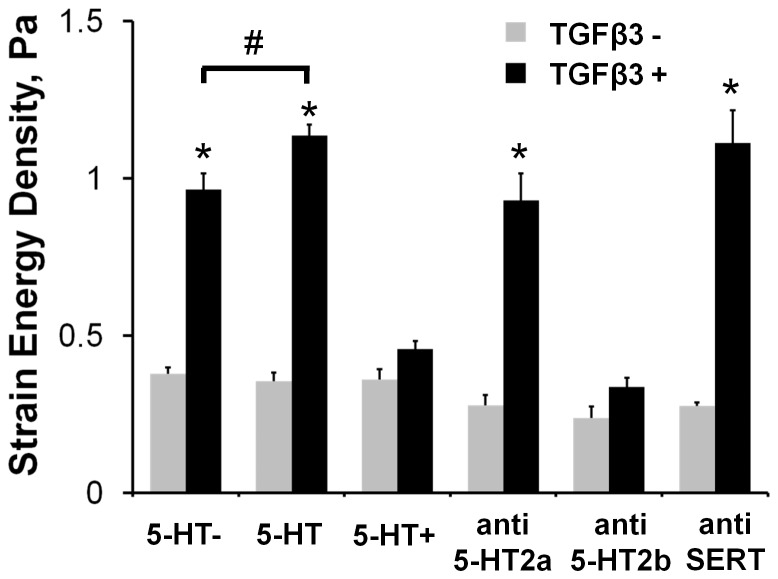
5-HT signaling modulates TGFβ3 induced AV cushion stiffness. Physiological dosages of 5-HT (470 nM, *5-HT*) exacerbated TGFβ3 stiffening, while elevated dosages (47 µM, *5-HT+*) eliminated it. Molecular inhibition of the 5-HT2a receptor (MDL100907 10 nM, *anti-5-HT2a*) and the serotonin transporter (Fluoxetine 10 µM, *anti-SERT*) did not affect TGFβ3 mediated biomechanical stiffening. Inhibition of the 5-HT2b receptor (SB204741 35 µM, *anti-5-HT2b*) however eliminated the stiffening effect of TGFβ3. mean ± SEM, n≥6, *p<0.0001 t-test relative to control, #p<0.05 2-way ANOVA with Tukey post-hoc test.

The effect of 5-HT dose on biomechanical remodeling, independently and in combination with TGFβ3, was systematically evaluated through the stiffness and compaction metrics of the AV cushion organ culture system. 5-HT administration by itself had no statistically significant effect on cushion stiffness. Combined treatment of TGFβ3 with physiological 5-HT (470 nM) increased AV cushion stiffness (W_T+5-HT_ = 1.136±0.035), but high 5-HT dose (5-HT+ = 47 µM) eliminated any TGFβ3 induced stiffening effect (W_T+5-HT+_ = 0.457±0.025, Figure 4). Neither selective inhibition of the 5-HT2a (MDL100907 10 nM), 5-HT2b (SB204741 2.6 µM) receptors, nor the serotonin transporter SERT (Flouxetine 10 µM) alone affected cushion stiffness (Figure 4). Yet in combination with TGFβ3, the anti-5-HT2b treatment completely blocked TGFβ3 dependent stiffness and compaction behavior (Figure 4 & Figure S3). Inhibition of the 5-HT2a receptor or SERT had no measurable effect on TGFβ3 induced cushion biomechanics. The compaction and stiffness changes induced by 5-HT potentiated TGFβ3 followed the same trend of TGFβ3 treatment alone, with compaction decreasing as stiffness increased and vice versa (Figure 4 & Figure S3). The additional stiffening effect of 5-HT with TGFβ3 was also eliminated with Alk5 inhibition, as shown through the combined treatment of TGFβ3+5-HT+anti-Alk5 in Figure S4. This combined treatment generated a strain energy density similar to the TGFβ3+anti-Alk5 treatment (0.209±0.023 Pa vs 0.245±0.16 Pa, respectively), and further supported that the effects of 5-HT signaling on AV valve remodeling is dependent on canonical TGFβ signaling. Together, these findings suggest that exogenous 5-HT acts through the 5-HT2b receptor to augment or impair TGFβ3 induced cushion stiffening and compaction in a dose-dependent manner.

### 5-HT modulates TGFβ3 regulation of AV cushion mesenchyme phenotype

**Figure 5:**
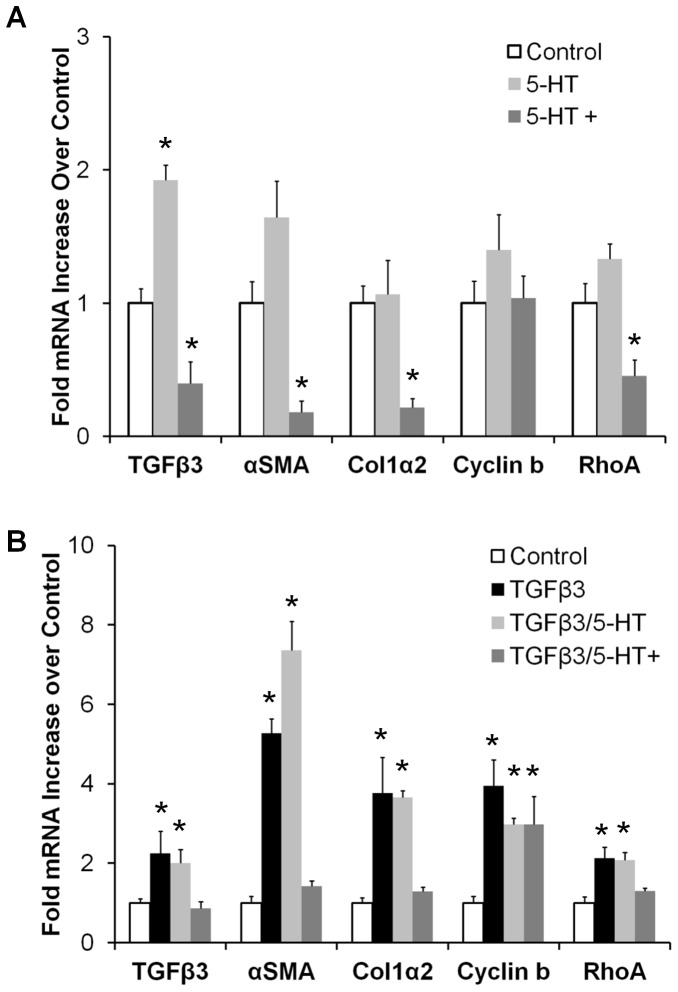
5-HT treatment modulates TGFβ3 mediated gene expression. **A**) TGFβ3 mRNA transcripts increase with physiological 5-HT (470 nM, *5-HT*), but decrease at high dose (47 µM, *5-HT+*). αSMA, RhoA, and col1α2, were not affected by physiological 5-HT dose, but were significantly downregulated with high 5-HT treatment. **B**) High 5-HT treatment mitigates exogenous TGFβ3 induced contractile gene expression, while TGFβ3 induced proliferation was independent of 5-HT dose. mean ± SEM, n = 3–5 pooled samples of 8–10 cushions, *p<0.05 via ANOVA comparisons with controls.

Exogenous 5-HT administration potentiated remodeling-relevant gene expression in organ cultured AV cushion mesenchyme. TGFβ3 mRNA transitioned from 1.9±0.1 fold upregulation over controls at physiological 5-HT to 0.40±0.16 downregulation at high 5-HT dose (Figure 5A). The physiological 5-HT dose had no statistically significant effect on αSMA, col1α2, cyclin b2, and RhoA expression. In contrast, high 5-HT significantly decreased transcription of αSMA (0.18±0.09), collagen1α2 (0.22±0.07), and RhoA (0.46±0.11 Figure 5A). No effect on cyclin b2 expression was observed at either dose, suggesting proliferation was not directly regulated by 5-HT. Physiological 5-HT did not affect TGFβ3 induced gene expression (Figure 5B), but high dose 5-HT markedly reduced gene expression of TGFβ3 (0.86±0.20 vs. 2.2±0.6), αSMA (1.4±0.4 vs. 5.3±0.4), collagen1α2 (1.3±0.3 vs. 3.8±0.9), and RhoA (1.3±0.2 vs. 2.1±0.3) (Figure 5B). Proliferation-related gene cyclin b2 was not significantly affected by 5-HT in combination with TGFβ3. These results suggest that exogenous 5-HT potentiates TGFβ3 more likely through interaction with upstream activation points and/or TGFβ3 synthesis, rather than by interacting with TGFβ3 downstream targets directly.

We also analyzed the mRNA expression of intracellular 5-HT (i5-HT) related genes transglutaminase 2 (TGM2) and SERT. i5-HT transamidates small GTPases and matrix proteins, in a process called “serotonylation” [44]. TGM2 is an i5-HT binding partner which assists transamidation of RhoA [55] and fibronectin [56], altering tissue mechanics through GTPase activation and matrix protein cross-linking, respectively. SERT mRNA expression was significantly increased with 5-HT treatment (1.5±0.2 fold, p<0.05), but was downregulated with the 5-HT+ dose (0.46±0.12 fold, Figure S5A). TGFβ3 treatment stimulated a 4.0±1.0 fold increase in TGM2, but SERT transcription remained near control levels (0.70±0.11, Figure S5B). Addition of 5-HT with TGFβ3 significantly decreased SERT and TGM2 mRNA, regardless of 5-HT dose. Although TGFβ3 treatment did upregulate TGM2, the downregulation of SERT by 5-HT treatment and the lack of mechanical changes seen with the SERT inhibitor suggest that serotonylation is not a primary mechanism of stiffness increase in the *ex vivo* culture remodeling results.

### Elevated 5-HT induces atrioventricular valvuloseptal defects in ovo

**Figure 6:**
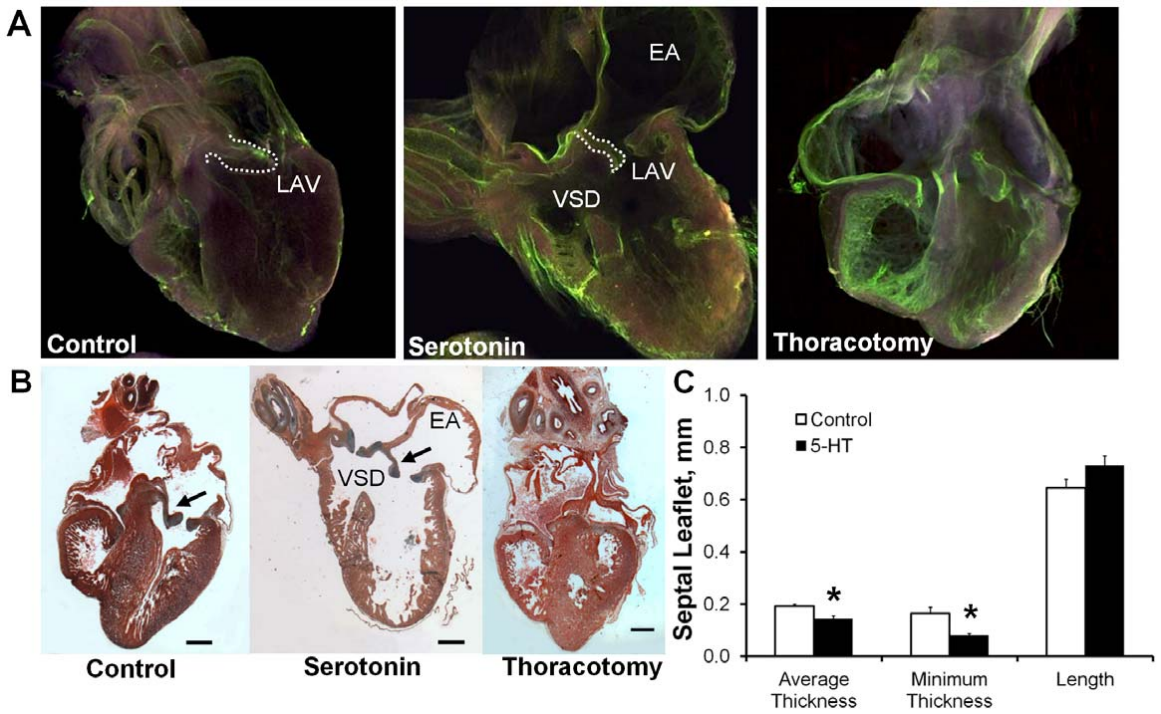
5-HT administration *in ovo* induces cardiac defects. **A**) Representative virtual sections of control, 5-HT treated, and thoracotomy sham control hearts at HH36 via endopainting and confocal microscopy. **B**) Representative Movat's pentachrome stained sections of hearts with the same conditions. Prominent cardiac defects, including enlarged atria (EA) and ventricular septal defect (VSD), were associated with malformed and malfunctioning AV valves (arrows). 25×, scale bar = 500 µm. **C**) Left septal leaflet average thickness and minimum thickness are both statistically thinner in 5-HT treated leaflets than control. mean ± SEM, n = 3–6 hearts per treatment, *p<0.05, t-test.

As the effects of TGFβ signaling on valve formation are well studied [17], [18], [57], we here test whether exogenous 5-HT administration *in ovo* alters valve morphogenesis. 5-HT administration *in ovo* at HH17 induced a spectrum of cardiac defects by HH36 (Day 10) as summarized in Table 1. Temporal and dosage dependant viability curves (Figure S6A) showed that a 0.7 mg dose was over 50% lethal at HH36, but administration of the same dose of 5-HT at HH25 or HH31 did not result in further lethality or defect formation (data not shown). The only gross malformations observed were localized to the heart and chest wall. Approximately 42% (24/57) of affected embryos exhibited an ectopic heart which protruded through an incomplete chest wall closure (Figure S6B). To confirm that interior defects resulted specifically from 5-HT exposure and not secondarily from the ectopia, an experimental thoracotomy was performed to model the ectopic condition. We found no statistically significant occurrence of any cardiac defects with experimental ectopia, supporting that 5-HT was responsible for the cardiac defects observed. A ventricular septal defect (VSD or SVSD) occurred in 42% (24/57) of the defective embryos. Approximately 18% (10/57) of the embryos exhibited double outlet right ventricle (DORV) defects. 5-HT administration also resulted in significantly enlarged atria with thinned walls in 35% (20/57) of the defective embryos (Table 1, Figure 6A). All of the embryos with DORV also exhibited highly stenotic or atretic atrioventricular (AV) valves (Figure 6B), with the normally muscular flap valve in the right AV canal appearing thin and fibrous like the left AV valve. Regardless of gross cardiac defect identified, the average (0.144±0.009 mm, mean ± SEM) and minimal (0.080±0.007 mm) thickness of the left AV septal leaflet was thinner in 5-HT treated embryos than controls (0.191±0.009 and 0.165±0.023 mm respectively, Figure 6C). No differences were found in mural leaflet thickness, or in the length of either leaflet. The reduction in AV valve thickness with 5-HT treatment indicated an increase in tissue compaction, and may possibly be a recapitulation of the migratory/contractile phenotype observed *ex vivo*.

**Table 1:** Cardiac Defect Summary of *in ovo* 5-HT Administration.

	Control	Serotonin	Thoracotomy^1^
# of embryos treated (HH17)	35	133	107
# of embryos survived (HH36)	34	60	49
# of defective embryos (HH36)	0	57*	27
Summary of Defects Represented in Survival Groups^2^			
Ectopic	-	24*	25*
VSD	-	5	-
SVSD	-	19*	1
DILV	-	3	-
DOLV	-	1	-
DORV	-	10*	-
Enlarged Atria	-	20*	3

1 Thoracotomy control of ectopic heart condition was created by mechanically debriding the chest dermis and pericardium at HH17.

2 Several embryos possessed more than one defect. VSD – ventricular septal defect; SVSD – *stenotic* VSD; DILV – double inlet left ventricle; DOLV – double outlet left ventricle; DORV – double outlet right ventricle.

* p<0.05 Chi-Squared test.

### Exogenous 5-HT increases AV cushion stiffness through TGFβ signaling in ovo

**Figure 7:**
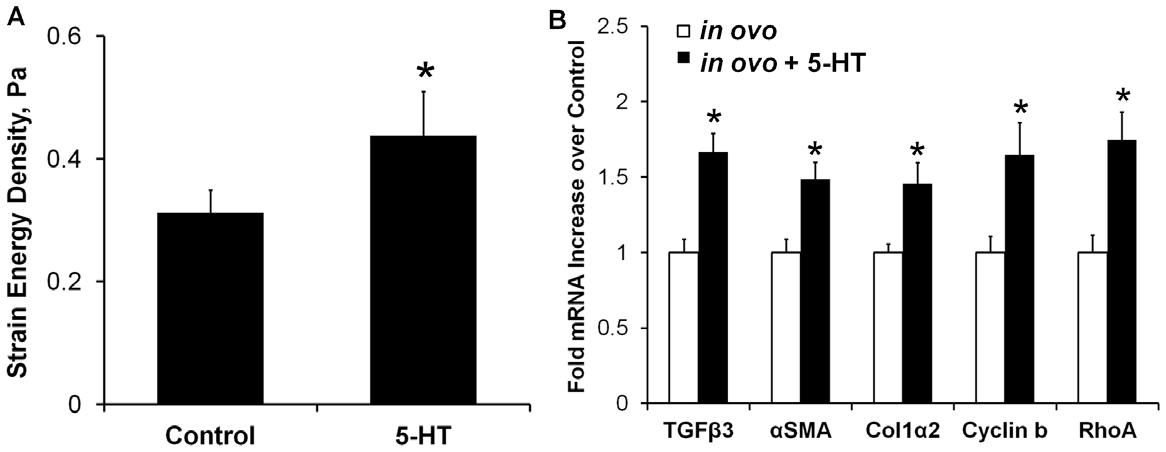
Exogenous 5-HT increases AV cushion stiffness and TGFβ related remodeling genes *in ovo*. **A**) The strain energy density (Pa) of HH25 cushions increased 1.4 fold with systemic 5-HT treatment *in ovo*, mean ± SEM, n = 8–10 cushion, *p<0.05, t-test. **B**) Gene expression levels of HH25 AV cushions isolated from embryos treated with 5-HT at HH17 (48 hours). mean ± SEM, n = 6–10 samples, each of 8–10 pooled HH25 cushions, *p<0.05, t-test.

**Figure 8:**
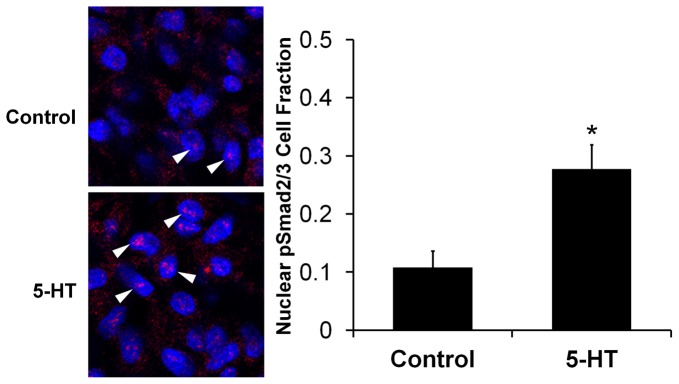
5-HT increases AV cushion pSmad2/3 expression *in ovo*. **A**) Representative images of pSmad2/3 staining. Arrows indicate pSmad2/3 positive cells. Cell nuclei – blue, pSmad2/3 – red. **B**) Embryos treated with systemic 5-HT at HH17 have increased pSmad2/3 expression at HH25 indicating elevated TGFβ signaling. n = 6, mean ± SEM *p<0.01, t-test.

Systemic 5-HT treatment at HH17 resulted in a statistically significant 1.4±0.2 fold increase in AV cushion stiffness over control at stage HH25 (strain energy density of 0.43±0.06 Pa vs. 0.31±0.03 Pa, *p<0.05, Figure 7A). We next analyzed the mesenchymal gene expression patterns in this *in ovo* system. 5-HT significantly upregulated TGFβ3 (1.7±0.1), αSMA (1.5±0.1), col1α2 (1.5±0.1), cyclin b (1.6±0.2), and RhoA (1.7±0.2) (*p<0.05, Figure 7B). Interestingly, the TGFβ3 mRNA expression was comparable to that observed in the *ex ovo* organ culture treatment of TGFβ3 alone (2.2±0.6), 5-HT alone (1.9±0.1), and TGFβ3+5-HT (2.0±0.3). αSMA and col1α2 mRNA were also upregulated *in ovo* with 5-HT, but less than with direct TGFβ3 administration in *ex vivo* culture (αSMA – 1.5 vs 5.7, RhoA – 1.7 vs 2.1). The similar mRNA profiles of the candidate genes in both models suggested that 5-HT also potentiates TGFβ signaling in AV cushions *in ovo*. To confirm that the 5-HT treatment was indeed modulating TGFβ signaling activity *in ovo*, we quantified nuclear pSmad2/3 expression in HH25 cushions with and without 5-HT treatment (Figure 8). 5-HT treatment increased the number of cell nuclei with localized pSmad2/3 expression 2.6±0.8 fold over control embryos (0.28±0.04 vs. 0.11±0.03, p<0.01). Together these results demonstrate that 5-HT potentiates TGFβ signaling in AV cushions to control contractile differentiation, proliferation, and biomechanical remodeling.

## Discussion

In this study we implemented a quantitative organ culture assay that simultaneously interrogated the contributions of cellular and molecular signaling to drive cushion tissue-level remodeling and biomechanical strengthening. TGFβ3 stimulated a 2.5 fold increase in biomechanical stiffness (Figure 1), generated in part by an increase in cell traction. This contractile phenotype is a common outcome of TGFβ signaling in post-natal valve tissue. For instance, porcine aortic valves express contractile marker αSMA when stimulated by TGFβ1 *in situ* [58]. Porcine aortic valve interstitial cells (VICs) embedded in collagen gels expressed αSMA in response to TGFβ1, and demonstrated significant gel compaction over untreated gels [59]. Similarly, TGFβ3 treated embryonic AV progenitors compacted collagen gels to 10% of initial area [23]. Yet in contrast to these reports, TGFβ3 induced contractility did not result in hyper-compacted AV cushions (Figure 2), but instead compacted less than controls. A key distinction between these two assays is that *in vitro* collagen gel cultures have much lower cell densities than our *ex vivo* system. The effect of proliferation on volume change is virtually undetectable in these gels, and cell traction dominates the compaction behavior. In native tissues, especially in the embryo, changes in cell proliferation and/or apoptosis have a significant impact on resulting tissue volume and apparent compaction. Hanging-drop culture of AV cushions enables precise control of the biochemical environment while maintaining the natural structural and cellular composition of the cushion. The lack of compaction with TGFβ3 treatment is therefore most likely due to a counterbalancing from increases in cell proliferation (Figure 3B) and ECM synthesis. This supports a mechanism of simultaneous tissue growth, matrix reorganization, and biomechanical stiffening during embryonic valve formation that is driven by a complex coordination of cell tractions, matrix synthesis, and cell proliferation. These findings underscore that embryonic valve mechanics, which is critical for proper valve function, cannot be inferred strictly from isolated compaction, proliferation, or matrix synthesis data, but is best measured directly.

The interplay of TGFβ3 and 5-HT signaling was most notably seen through the potentiation of TGFβ3 gene expression by 5-HT dose (Figure 5). The physiological 5-HT concentration upregulated TGFβ3 expression, while the high concentration downregulated expression. Upregulation of TGFβ expression by 5-HT has been observed in several cardiac cells and tissues, though the molecular mechanism is still unclear. Adult aortic valve interstitial cells treated with 5-HT have increased TGFβ1 activity, predominantly through the 5-HT2a receptor [25], [60]. Neo-natal rat cardiac fibroblasts treated with 5-HT and 5-HT2a agonists upregulated αSMA protein expression, which is a marker for fibroblast differentiation and a gene induced by TGFβ signaling [61]. Similarly, TGFβ1 and αSMA expression were elevated in SERT cre-lox KO mice hearts through heightened 5-HT2a signaling in late embryonic stage mice, purportedly due to excess 5-HT from SERT inhibition [43]. Other reports point to 5-HT2b as the key mechanism. 5-HT administration in adult rats increased 5-HT2b mRNA expression in both aortic and mitral valves, demonstrating a positive response to 5-HT treatment [50]. SERT mRNA was downregulated in these valves denoting a negative response to elevated 5-HT, which our results also demonstrate (Figure S5B). The 5-HT2b receptor, TGFβ receptor type I and II, and the TGFβ latent binding protein were all more expressed in canine myxomatous mitral valves than normal valves, suggesting a coupling of these two pathways through 5-HT2b [26]. Long-term 5-HT treatment of rats generated valve-related echocardiographic and histology defects [62], but these defects did not occur in rats simultaneously treated with a 5-HT2b inhibitor [51]. This suggests that the 5-HT2b receptor may be a key pathway for cardiac and valve tissue remodeling. Cardiac fibroblast studies indicate that 5-HT upregulates TGFβ1 through a mutual transactivation of the epidermal growth factor (EGF) pathway and the 5-HT2b receptor [45], [63]. Our results support a 5-HT2b dependant mechanism, as seen by 5-HT2b inhibition effectively blocking TGFβ3 stiffening. The TGFβ stiffening effect was independent of 5-HT2a and SERT. Although TGFβ3 upregulated TGM2 expression, 5-HT treatment mitigated this expression which suggests TGM2 activity does not contribute to the enhanced stiffening of TGFβ/5-HT signaling. High 5-HT also mitigated TGFβ3 stiffening, which may be due to desensitization of the 5-HT2b receptor by sustained high 5-HT exposure. 5-HT increased pSmad2/3 phosphorylation in cushion mesenchyme *in ovo*. This suggests that 5-HT signaling through 5HT2b may interact with Smad2/3 signaling, but further studies are warranted to clarify potential roles of other intermediate or downstream targets.

In our *in ovo* model, systemic 5-HT elevation induced severe heart defects, including failure of the ventricular septum to close, ballooned atria, DORV, and hyper-contracted AV valves. Variations of these defects have been observed in other TGFβ and 5-HT related studies. VSDs are the most prevalent congenital heart defects observed, occurring in approximately 50% of all clinical cardiac malformations [64], [65]. Selective serotonin uptake inhibitors (SSRI) taken during the first trimester of pregnancy were associated with a statistical increase in VSD prevalence in newborns [66]. Our data supports elevated extracellular 5-HT as a possible cause of this correlation. Removal of TGFβ secondary messenger Smad4 causes VSDs and other lethal congenital defects, which are presumed to be the consequence of decreased TGFβ signaling [67]. Yet removal of TGFβ inhibitory messenger Smad7 also generates VSDs [68], indicating that exacerbated TGFβ signaling can also generate significant cardiac defects. The dilated atria observed in our model are not explicitly reported in other 5-HT studies, suggesting the defect may result from secondary effects, such as altered hemodynamics from valve incompetence. For instance, enlarged atria have been induced in zebrafish embryos through mechanical obstruction of the AV canal [69]. Our avian model exhibited a small (18%), but statistically significant, penetrance of DORV, which is a predominant congenital defect in TGFβ2 KO mice (87% penetrance) [17]. Collectively these defects highlight the morphogenetic potential of 5-HT in early cardiac development, and the similar spectrum of defects generated across 5-HT and TGFβ related animal models.

An interaction of TGFβ and 5-HT signaling was observed *in ovo* through the upregulation of TGFβ3 and contractile genes in the AV cushions (Figure 7B), the increase in pSmad2/3 expression (Figure 8), and the resulting thinned valve morphology (Figure 6C). While the pSmad2/3 and mRNA expression confirms that aspects of the *ex vivo* results occur *in ovo*, it is unclear whether elevated TGFβ signaling at HH25 is solely responsible for the thinned valve morphology observed at HH36. Hyperplastic and thickened AV valves occur in TGFβ2 KO (31% penetrance) [17], [70], and TGFβ latent binding protein KO (81% penetrance) [57] animals, which supports this hypothesis. However, systemic 5-HT administration in adult rats generates thickened valves, with treatment duration dependent remodeling. Subcutaneous 5-HT injections for 7 days in adult rats produced thickened AV valves rich in GAGs [50], while 3-month treatment increased valve thickness, but consisted primarily of collagen [62]. Thickened, collagen-rich valves are also reported in adult SERT KO mice [71], and at late embryonic stage SERT KO pups [43]. Together these results indicate that elevated 5-HT signaling can instigate valvular remodeling *in vivo*, but changes in valve microstructure and morphology are clearly dependent on other factors such as treatment duration, specimen age, or secondary effects from accompanying congenital malformations. Altered hemodynamic loading can also generate defects, as evidenced through the serious malformations stimulated by mechanical perturbation [72], [73]. Yet hemodynamic loading is simultaneously a consequence and stimulant of molecular signaling, interacting in a cyclical rather than a linear cause-effect manner. This again emphasizes the importance of direct assessment of mechanical stiffness, because it can distinguish the influence of these microstructure and microenvironment variations on valve performance.

Embryonic valve formation and maturation utilizes multiple TGFβ isoforms in spatially and temporally restricted ways that are also somewhat different between species [16], [18]. We chose to focus on TGFβ3 over either TGFβ1 or TGFβ2 because of its principal role in cell invasion during chick cushion EMT [16], and confirmed increase in expression during post-EMT [23]. Our results establish a molecular mechanism for short-term (24 hours) TGFβ3 stimulation on AV cushion biomechanical remodeling, but the effects of prolonged signaling on biomechanical and morphological changes remain unclear. This could be addressed with a combined *in vivo*/*in vitro* experimentation over more time points using a system like the approach presented here. The *ex vivo* culture system contains both endocardial and mesenchymal cells, but the lack of chick reactive antibodies prohibited the determination of cell specific responses. Our *in ovo* exogenous 5-HT administration model data complements existing data on genetic mutant animal models of TGFβ and 5-HT related signaling in cardiac development [17], [74]. Future studies will need to investigate whether the serotonin effects of TGFβ3 change with TGFβ3 dose.

In conclusion, tissue mechanics, cell phenotype, and molecular signaling all simultaneously direct and control tissue morphogenesis. Our results suggest that TGFβ is a potent stimulator of cushion stiffening, and that 5-HT is a key regulator of this stimulating effect. Connecting signaling networks with cell and tissue level responses will become increasingly important for understanding post-EMT valve remodeling and potentially other embryonic remodeling events. The quantitative experimental systems presented herein are an attractive approach for elucidating these multi-scale mechanisms and their downstream consequences.

## Supporting Information

Table S1 **RT-PCR Primer Sequences.**
(TIF)

Figure S1 **Minimal ECM organization in HH25 cushion supports use of an isotropic mechanical testing technique.** **A**) Confocal image of a HH25 cushion with ECM labeled via 5-DTAF protein stain at 10× magnification. **B**) 40× magnification. Note the lack of matrix fiber density or preferential fiber orientation at this stage of development.
(TIF)

Figure S2 **Compaction-related stiffness control.** **A**) Molecular inhibition of actin polymerization (Cytochalasin D, 1 µM) caused an 80–85% reduction in effective modulus. mean ± SEM, n≥6 *p<0.0001, t-test **B**) Cushion area increased with actin inhibition, resulting in a 3 fold decrease in measured compaction compared to control. Insets: Representative images of AV cushions before and after treatment, scale bar = 100 µm. mean ± SEM, n≥12, *p<0.0001, t-test.
(TIF)

Figure S3 **TGFβ3-induced decrease in compaction was blocked through inhibition of Alk5 (SB431542, 2.6 µM) or 5-HTR2b (SB204741 35 µM,** ***anti-5-HT2b*** **).** Neither 5-HTR2a inhibitor (MDL100907 10 nM, *anti-5-HT2a*) nor serotonin transporter inhibitor (Fluoxetine 10 µM, *anti-SERT*) affected TGFβ3 compaction behavior. mean ± SEM, n≥7, *p<0.05, t-test with respect to untreated controls.
(TIF)

Figure S4 **TGFβ3 and 5-HT stiffness generation is dependent on Alk5 signaling pathway.** Strain energy density (Pa) of cushions treated with TGFβ3 (1 ng/ml) only, TGFβ3+Alk5 inhibitor (SB431542, 2.6 µM anti-Alk5), TGFβ3+5-HT (470 nM), and TGFβ3+5-HT+anti-Alk5. mean ± SEM, n≥8. Different letter pairings denotes statistically significant p<0.05, 2-way ANOVA.
(TIF)

Figure S5 **Intracellular 5-HT uptake is modulated by 5-HT dose.** **A**) 5-HT transporter (SERT) gene expression was downregulated via high 5-HT (47 µM, *5-HT+*) dose, while transglutaminase 2 (TGM2) was not affected. The physiological dose of 5-HT (470 nM, *5-HT*) had no effect on either SERT or TGM2 gene expression. **B**) TGFβ3 (1 ng/ml) stimulated 4-fold increase in TGM2, which was mitigated by either doses of 5-HT. TGFβ3 had no effect on SERT expression. mean ± SEM, n = 3–4, *p<0.05, t-test.
(TIF)

Figure S6 **Characterization of** ***in ovo*** **5-HT administration model.** **A**) Plot of avian embryo viability as a function of time and 5-HT dose. 5-HT administration to the surface of HH17 chick embryos resulted in greater than 70% lethality at dosages above 0.75 mg. The majority of deaths occurred within 48 hours of incubation. Doses of 0.5 mg and below were over 80% viable with virtually no morphological defects. Doses administered at later incubation times (Day 5, Day 7) did not result in lethality or defects by HH36 (data not shown). 5-HT administration at the predicted 50% lethality dose (0.7 mg/100 µL) resulted in 55% lethality by Day 10. **B**) Representative image of ectopic heart (arrow) and unclosed chest (dashed line) observed with both 5-HT treatment and thoracotomy sham controls.
(TIF)
