# Correction: Serotonin Potentiates Transforming Growth Factor-beta3 Induced Biomechanical Remodeling in Avian Embryonic Atrioventricular Valves

**DOI:** 10.1371/annotation/7fdd5c53-e8ab-40b5-b297-e82c06a06e70

**Published:** 2012-10-23

**Authors:** Philip R. Buskohl, Michelle L. Sun, Robert P. Thompson, Jonathan T. Butcher

The second author's name was spelled incorrectly. The correct name is: Michelle J. Sun.

The correct citation is: Buskohl PR, Sun MJ, Thompson RP, Butcher JT (2012) Serotonin Potentiates Transforming Growth Factor-beta3 Induced Biomechanical Remodeling in Avian Embryonic Atrioventricular Valves. PLoS ONE 7(8): e42527. doi:10.1371/journal.pone.0042527 

