# Supplementary figures and images for: Serotonin Potentiates Transforming Growth Factor-beta3 Induced Biomechanical Remodeling in Avian Embryonic Atrioventricular Valves
Source: PLoS One. 2012 Aug 6;7(8):e42527. doi: 10.1371/journal.pone.0042527 (PMC3412853; doi:10.1371/journal.pone.0042527)

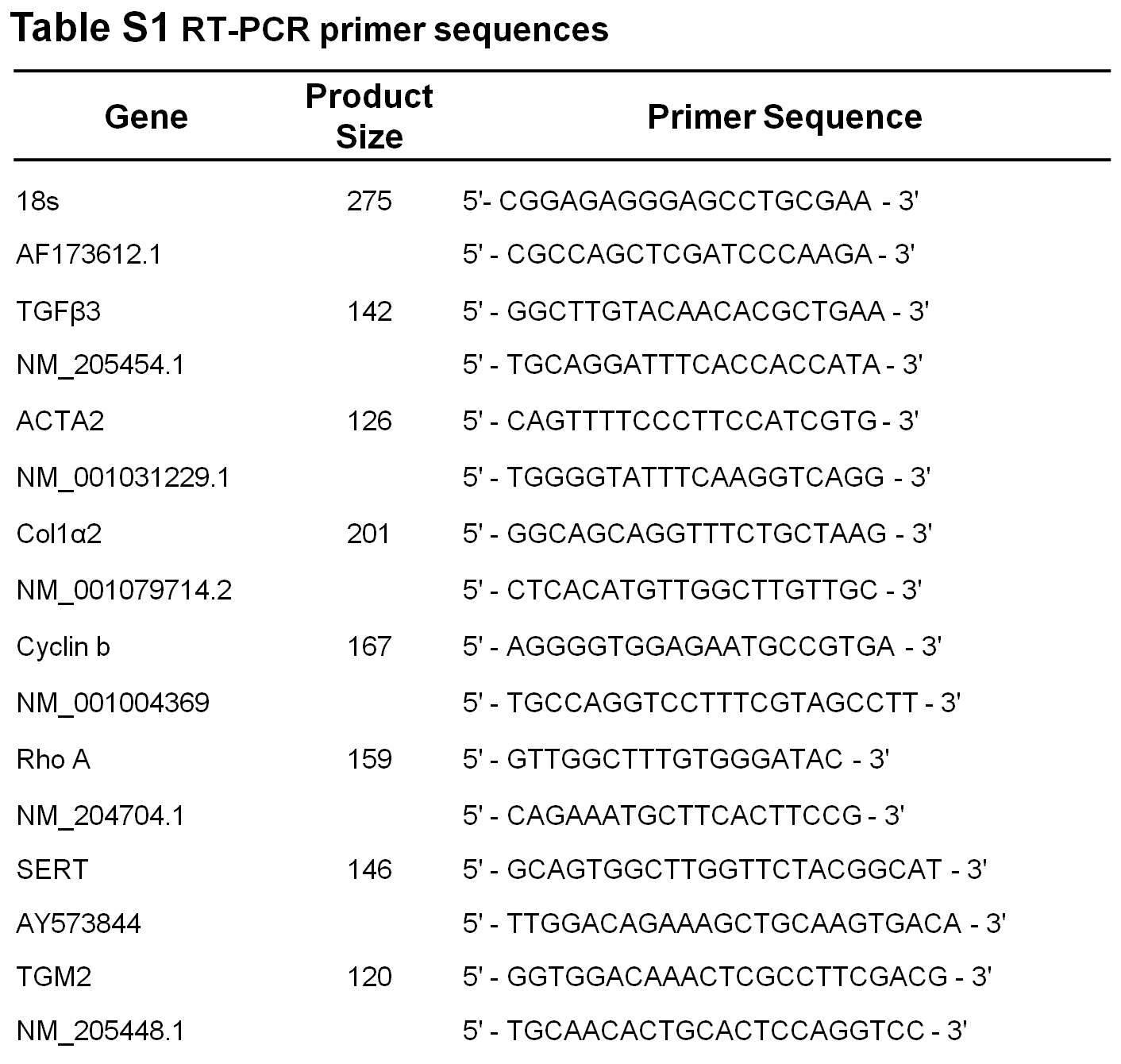

Supplement: Table S1 — RT-PCR Primer Sequences. (TIF) [file pone.0042527.s001.tif]

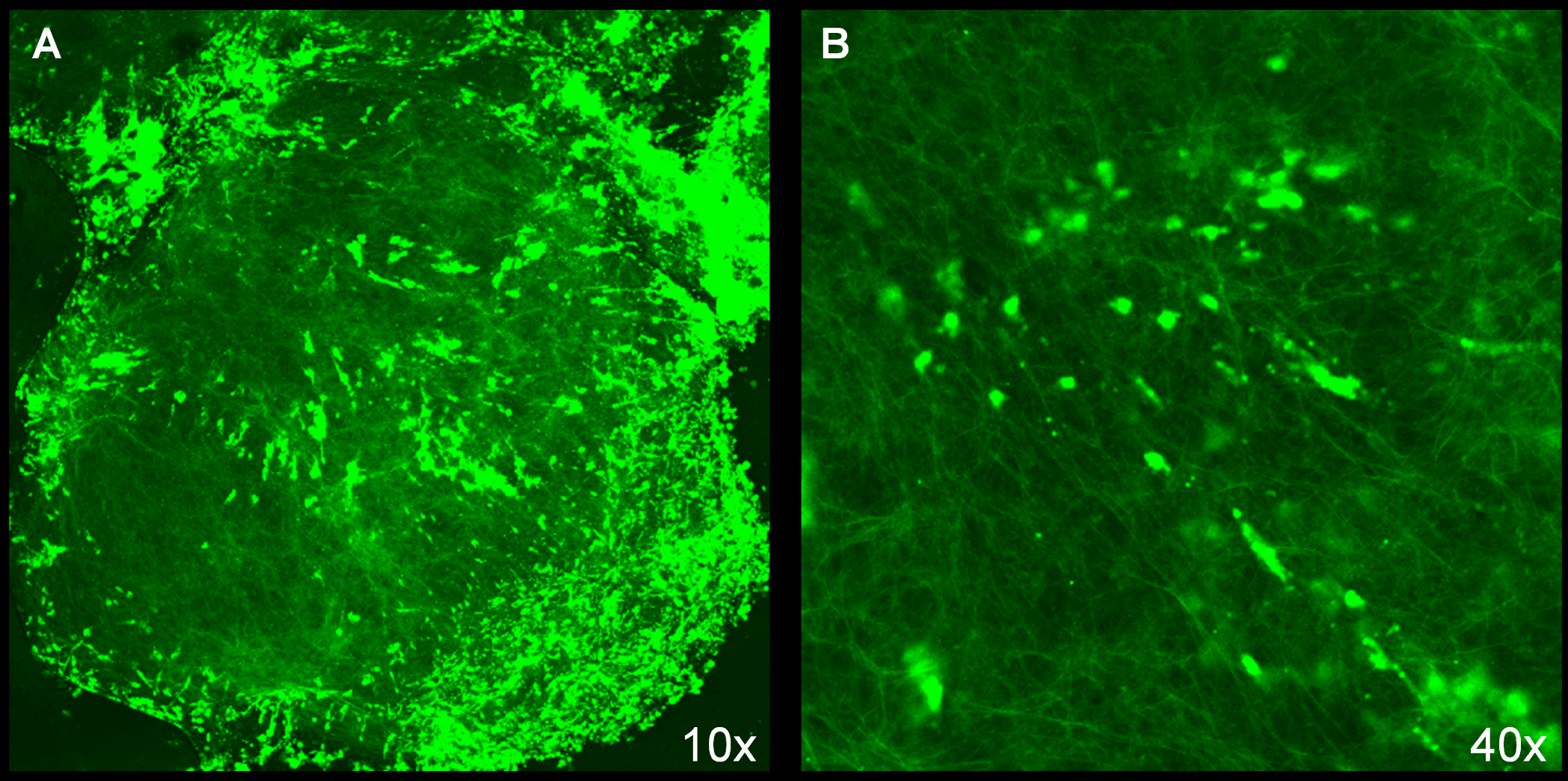

Supplement: Figure S1 — Minimal ECM organization in HH25 cushion supports use of an isotropic mechanical testing technique. A) Confocal image of a HH25 cushion with ECM labeled via 5-DTAF protein stain at 10× magnification. B) 40× magnification. Note the lack of matrix fiber density or preferential fiber orientation at this stage of development. (TIF) [file pone.0042527.s002.tif]

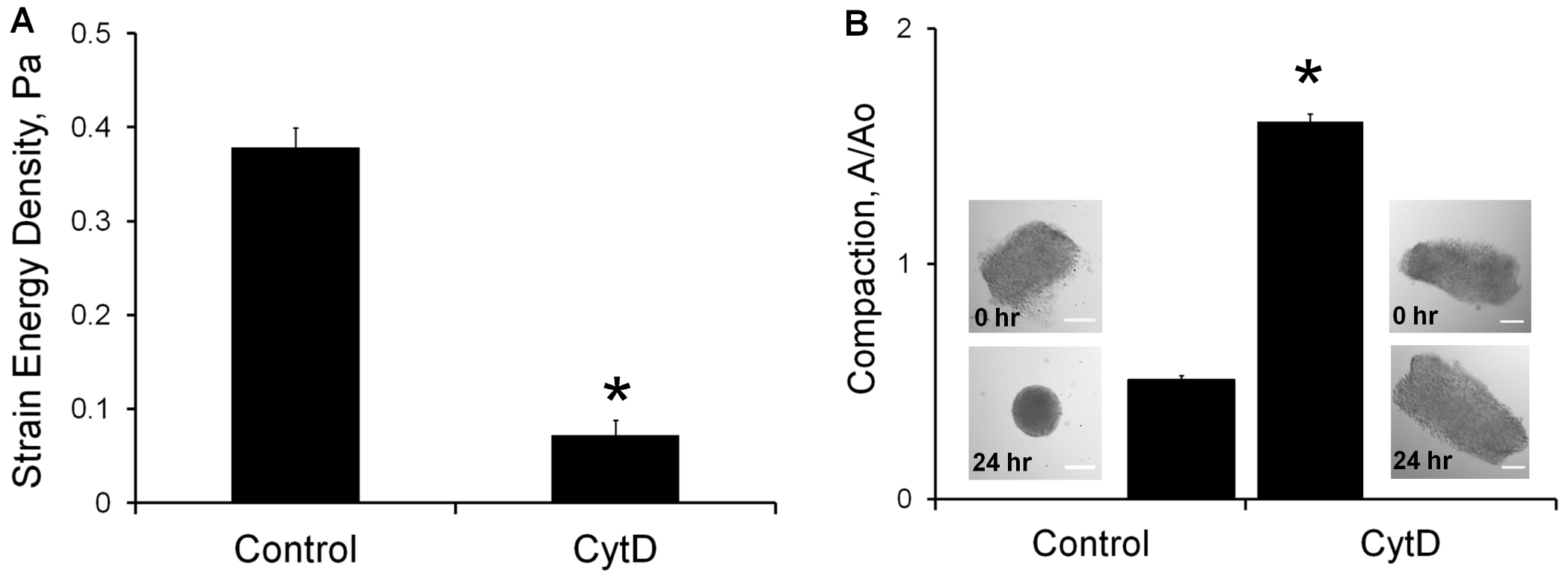

Supplement: Figure S2 — Compaction-related stiffness control. A) Molecular inhibition of actin polymerization (Cytochalasin D, 1 µM) caused an 80–85% reduction in effective modulus. mean ± SEM, n≥6 *p<0.0001, t-test B) Cushion area increased with actin inhibition, resulting in a 3 fold decrease in measured compaction compared to control. Insets: Representative images of AV cushions before and after treatment, scale bar = 100 µm. mean ± SEM, n≥12, *p<0.0001, t-test. (TIF) [file pone.0042527.s003.tif]

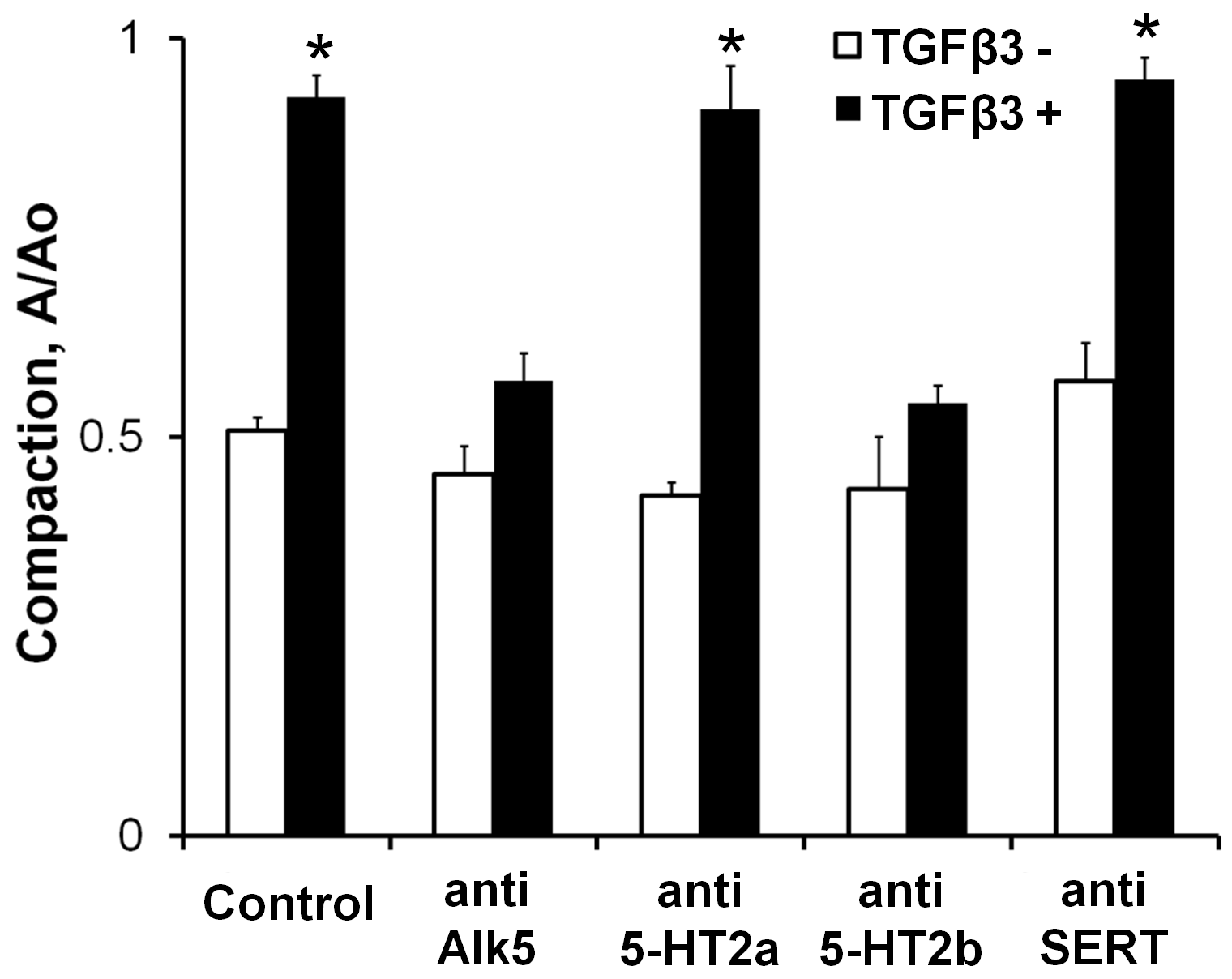

Supplement: Figure S3 — TGFβ3-induced decrease in compaction was blocked through inhibition of Alk5 (SB431542, 2.6 µM) or 5-HTR2b (SB204741 35 µM, anti-5-HT2b ). Neither 5-HTR2a inhibitor (MDL100907 10 nM, anti-5-HT2a) nor serotonin transporter inhibitor (Fluoxetine 10 µM, anti-SERT) affected TGFβ3 compaction behavior. mean ± SEM, n≥7, *p<0.05, t-test with respect to untreated controls. (TIF) [file pone.0042527.s004.tif]

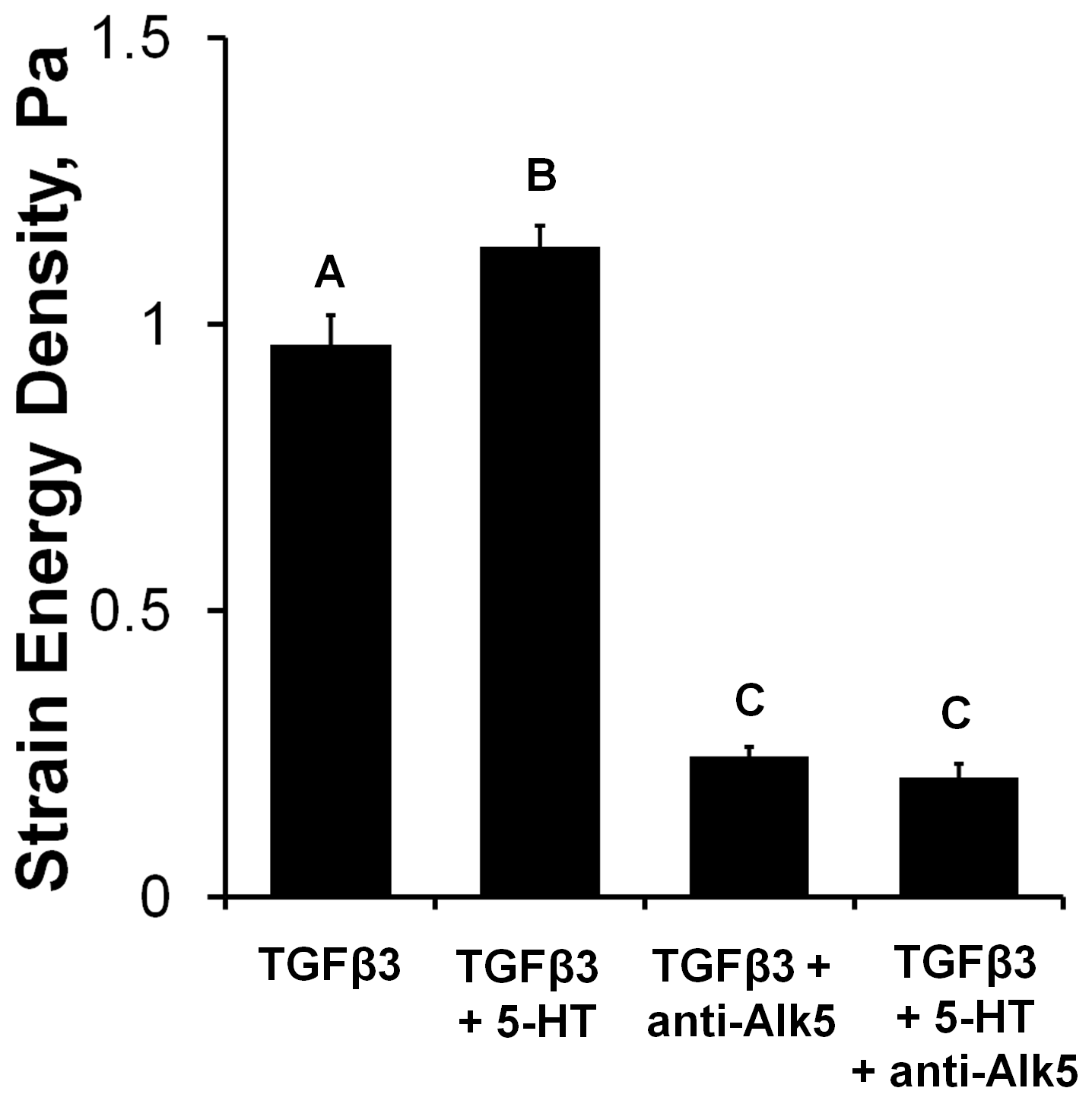

Supplement: Figure S4 — TGFβ3 and 5-HT stiffness generation is dependent on Alk5 signaling pathway. Strain energy density (Pa) of cushions treated with TGFβ3 (1 ng/ml) only, TGFβ3+Alk5 inhibitor (SB431542, 2.6 µM anti-Alk5), TGFβ3+5-HT (470 nM), and TGFβ3+5-HT+anti-Alk5. mean ± SEM, n≥8. Different letter pairings denotes statistically significant p<0.05, 2-way ANOVA. (TIF) [file pone.0042527.s005.tif]

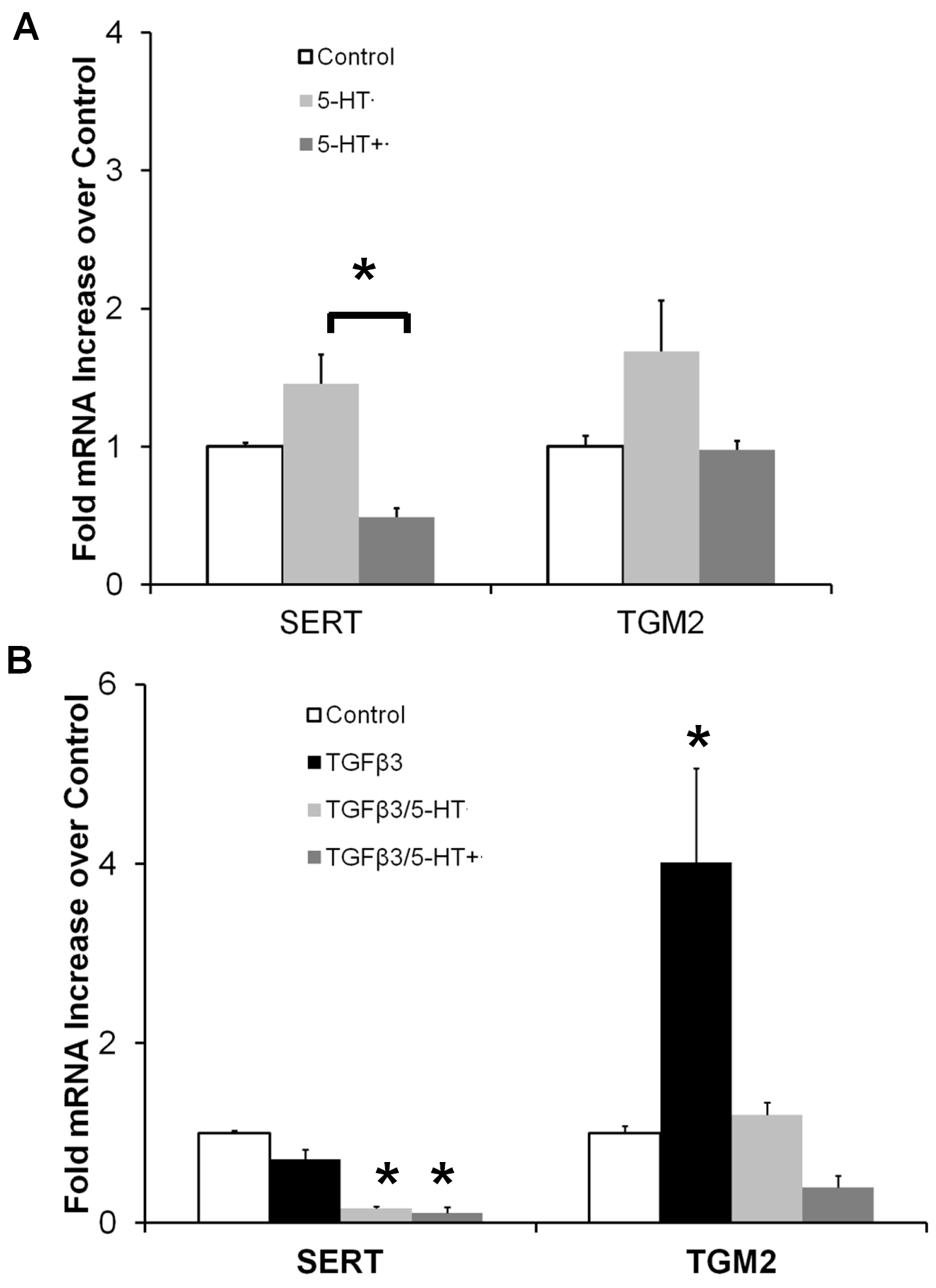

Supplement: Figure S5 — Intracellular 5-HT uptake is modulated by 5-HT dose. A) 5-HT transporter (SERT) gene expression was downregulated via high 5-HT (47 µM, 5-HT+) dose, while transglutaminase 2 (TGM2) was not affected. The physiological dose of 5-HT (470 nM, 5-HT) had no effect on either SERT or TGM2 gene expression. B) TGFβ3 (1 ng/ml) stimulated 4-fold increase in TGM2, which was mitigated by either doses of 5-HT. TGFβ3 had no effect on SERT expression. mean ± SEM, n = 3–4, *p<0.05, t-test. (TIF) [file pone.0042527.s006.tif]

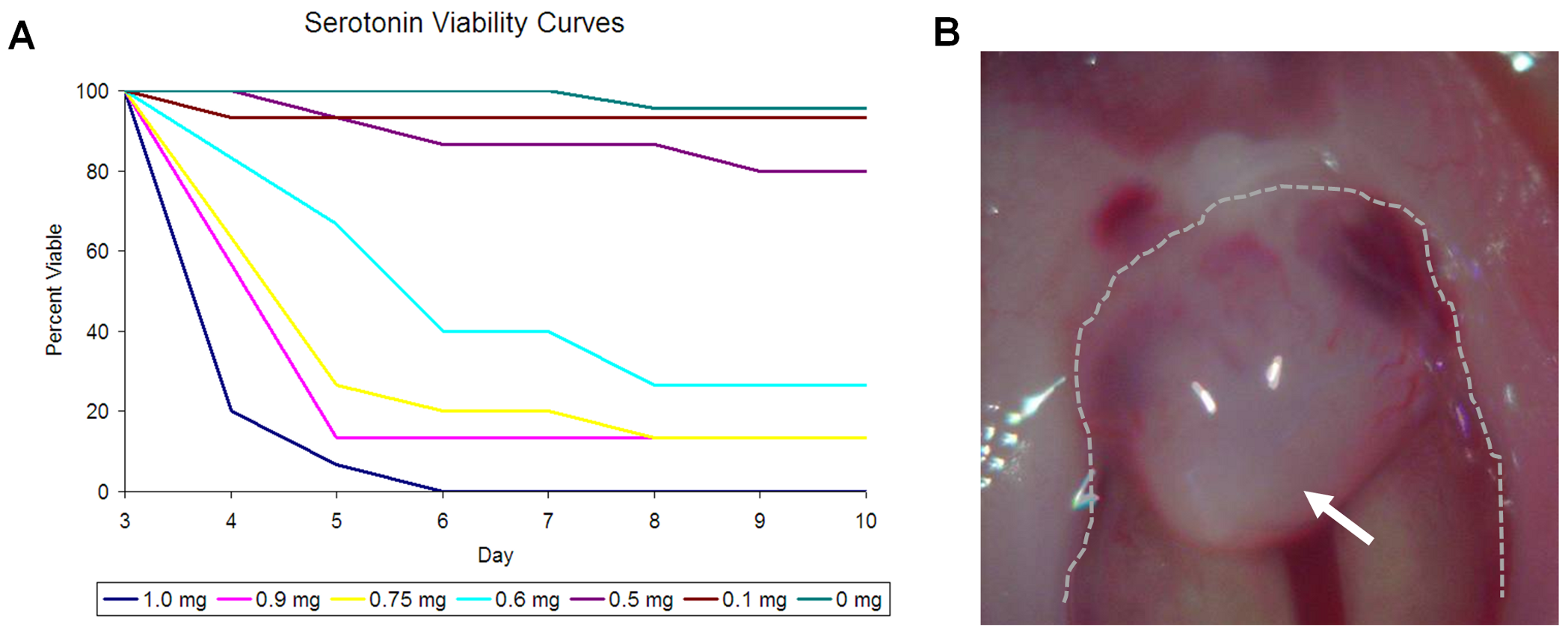

Supplement: Figure S6 — Characterization of in ovo 5-HT administration model. A) Plot of avian embryo viability as a function of time and 5-HT dose. 5-HT administration to the surface of HH17 chick embryos resulted in greater than 70% lethality at dosages above 0.75 mg. The majority of deaths occurred within 48 hours of incubation. Doses of 0.5 mg and below were over 80% viable with virtually no morphological defects. Doses administered at later incubation times (Day 5, Day 7) did not result in lethality or defects by HH36 (data not shown). 5-HT administration at the predicted 50% lethality dose (0.7 mg/100 µL) resulted in 55% lethality by Day 10. B) Representative image of ectopic heart (arrow) and unclosed chest (dashed line) observed with both 5-HT treatment and thoracotomy sham controls. (TIF) [file pone.0042527.s007.tif]
